# Disseminated extrapulmonary *Legionella pneumophila* infection presenting with panniculitis: case report and literature review

**DOI:** 10.1186/s12879-018-3378-0

**Published:** 2018-09-17

**Authors:** Maria N. Chitasombat, Natta Ratchatanawin, Yingluck Visessiri

**Affiliations:** 1 Division of Infectious Disease, Department of Medicine, Faculty of Medicine, Ramathibodi Hospital, Mahidol University, 270 Rama VI Road, Ratchathewi District, Bangkok, 10400 Thailand; 2 Division of Dermatology, Faculty of Medicine Ramathibodi Hospital, Mahidol University, Bangkok, Thailand; 3 Department of Pathology, Faculty of Medicine Ramathibodi Hospital, Mahidol University, 270 Rama VI Road, Ratchathewi District, Bangkok, Thailand

**Keywords:** *Legionella pneumophila*, Panniculitis, Lupus, Myositis, Myocarditis

## Abstract

**Background:**

Legionellosis is a well-known cause of pneumonia. Primary cutaneous and subcutaneous infection caused by *Legionella pneumophila* is rare and the diagnosis is challenging.

**Case presentation:**

A 38-year-old Thai woman with systemic lupus erythematosus and myasthenia gravis treated with prednisolone and azathioprine presented to our hospital with low-grade fever, diarrhea, and indurated skin lesions on both thighs. Initial examination showed plaques on both inner thighs. Magnetic resonance imaging showed myositis and swelling of the skin and subcutaneous tissue. Diagnosis of panniculitis due to *L. pneumophila* was carried out by histopathology, Gram stain, and 16S rRNA gene sequencing method of tissue biopsy from multiple sites on both thighs. Myocarditis was diagnosed by echocardiography. The final diagnosis was disseminated extrapulmonary legionellosis. Treatment comprised intravenous azithromycin for 3 weeks and the skin lesions, myositis and myocarditis resolved. Oral azithromycin and ciprofloxacin were continued for 3 months to ensure eradication of the organism. The patient’s overall condition improved.

**Conclusions:**

To our knowledge, we report the first case of *L. pneumophila* infection manifesting with panniculitis, possible myositis, and myocarditis in the absence of pneumonia. The diagnosis of extrapulmonary *Legionella* infection is difficult, especially in the absence of pneumonia. A high index of suspicion and appropriate culture with special media or molecular testing are required. Initiation of appropriate treatment is critical because delaying therapy was associated with progressive infection in our patient.

**Electronic supplementary material:**

The online version of this article (10.1186/s12879-018-3378-0) contains supplementary material, which is available to authorized users.

## Background

*Legionella* is a well-known cause of pneumonia. Extrapulmonary manifestations of Legionnaires’ disease include myocarditis [1, 2], neurological involvement (acute disseminated encephalomyelitis), and multiorgan failure [3, 4]. Legionnaires’ disease has been reported together with several types of skin lesions such as maculopapular rash, petechial rash, erythema with focal blister, cellulitis, pustules, abscesses, and subcutaneous masses [5]. Various species of *Legionella*, including *L. pneumophila, L. micdadei, L. cincinnatiensis, L. maceachernii,* and *L. feeleii*, cause skin/soft tissue infection, mostly among immunocompromised patients with pneumonia [6]. Primary cutaneous infection is a rare distinctive feature of direct inoculation of *Legionella* into skin and soft tissue, which occurs as cellulitis necrotizing fasciitis, as a postoperative complication [6–9]. The diagnosis of legionellosis can be challenging in the absence of pneumonia. To the best of our knowledge, this is the first description of panniculitis due to *Legionella*. This report highlights the challenges, pitfalls and importance of diagnosing extrapulmonary *Legionella* infection.

## Case presentation

**Fig. 1:**
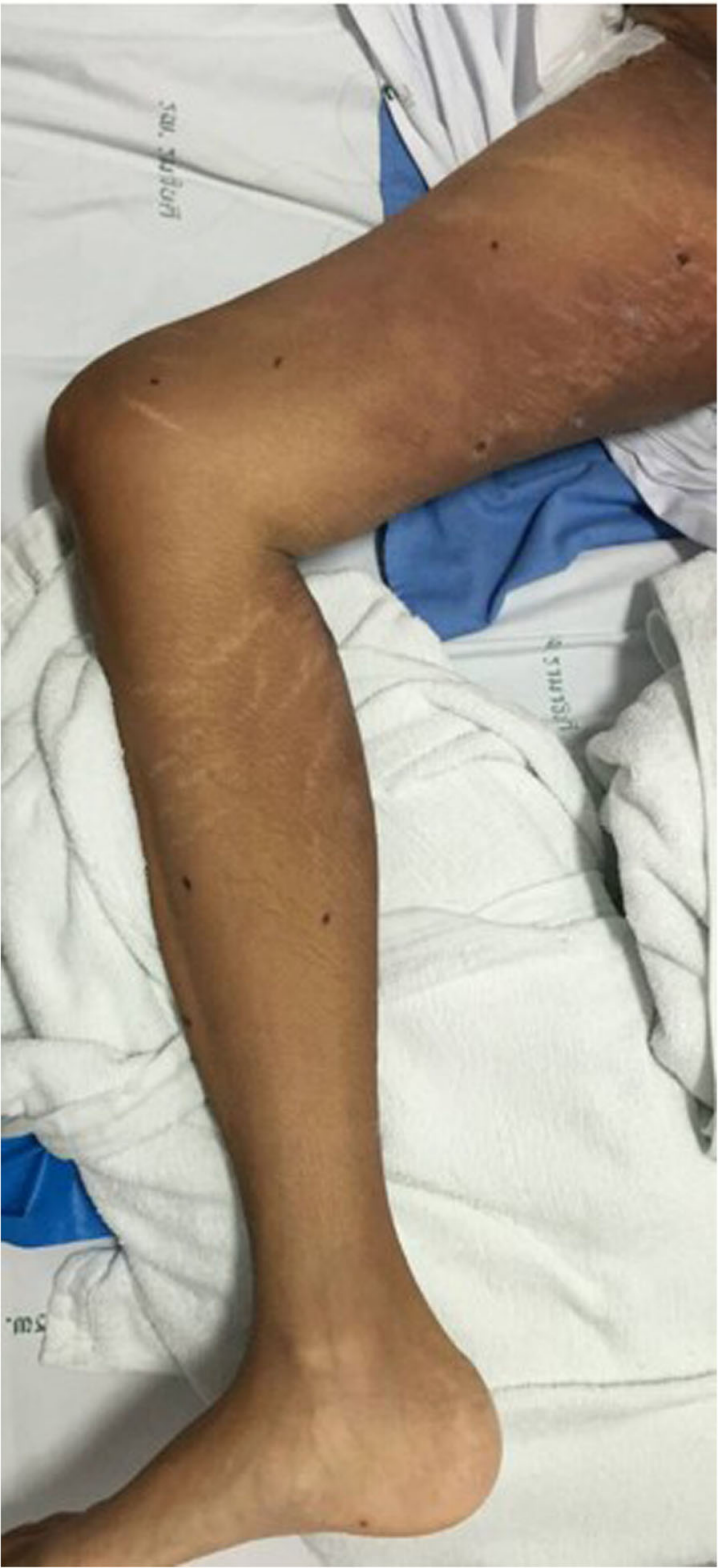
Multiple erythematous indurated plaques on the proximal right thigh, multiple healed crusted papules on the right lower extremity, and whitish striae (before treatment)

**Fig. 2:**
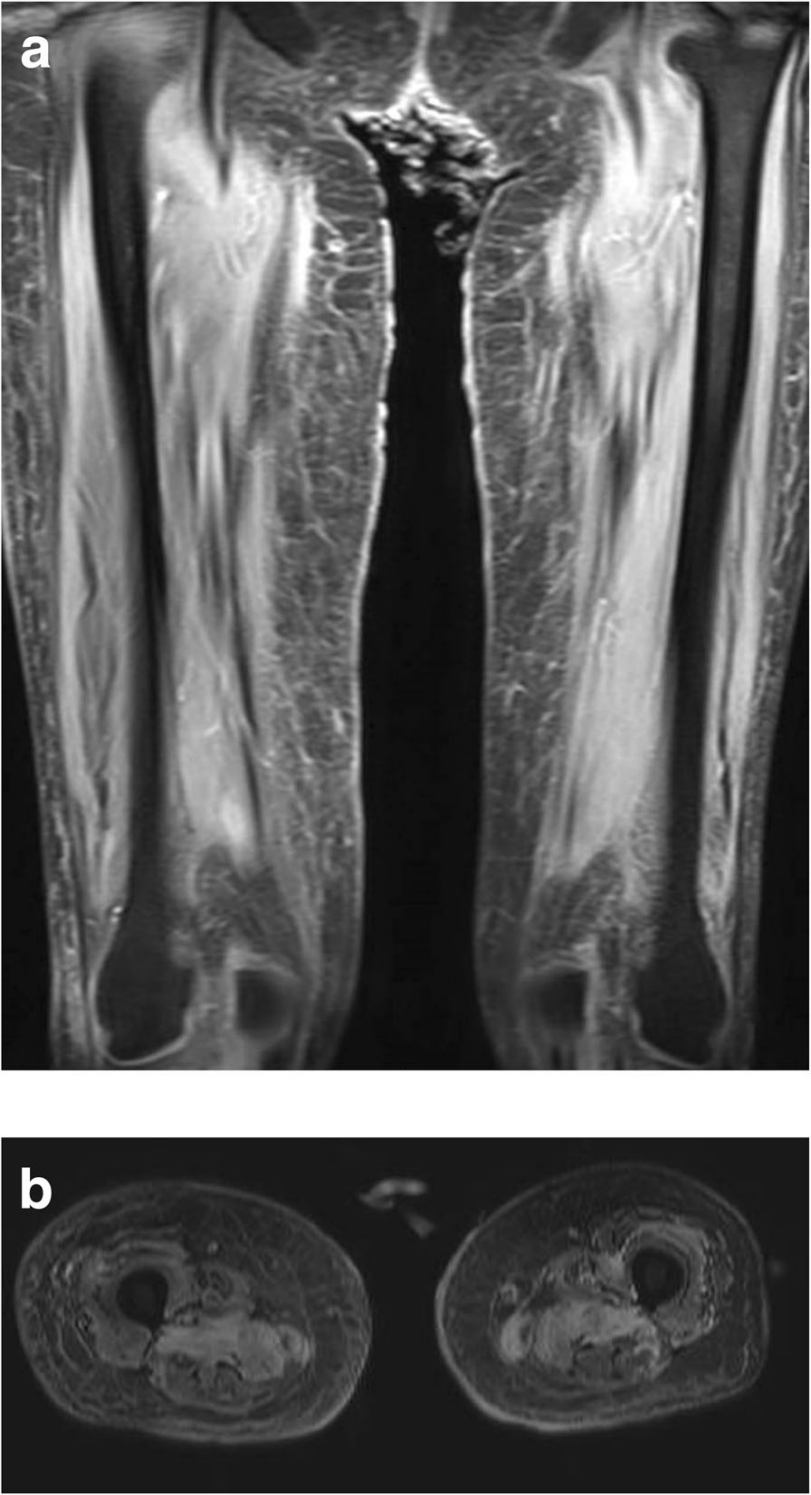
**a**. Coronal T2-weighted MRI of the thighs. **b**. Axial T2-weighted MRI of the thighs demonstrating enhancement of subcutaneous tissue and muscle. MRI, magnetic resonance imaging

**Fig. 3:**
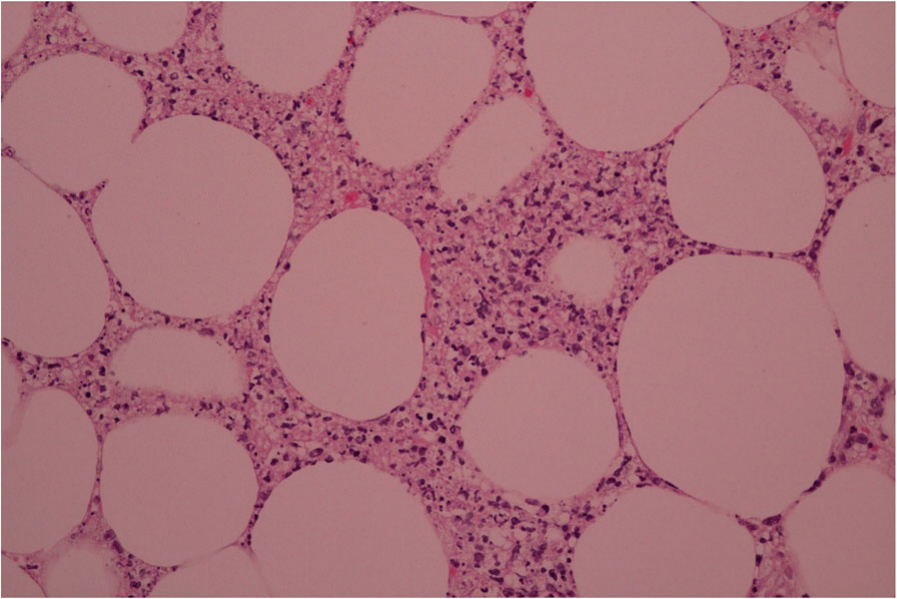
Histopathology of subcutaneous tissue of right thigh. Photomicrograph revealed neutrophil infiltration in the deep dermis to subcutaneous tissue and fat necrosis (200×, hematoxylin and eosin)

**Fig. 4:**
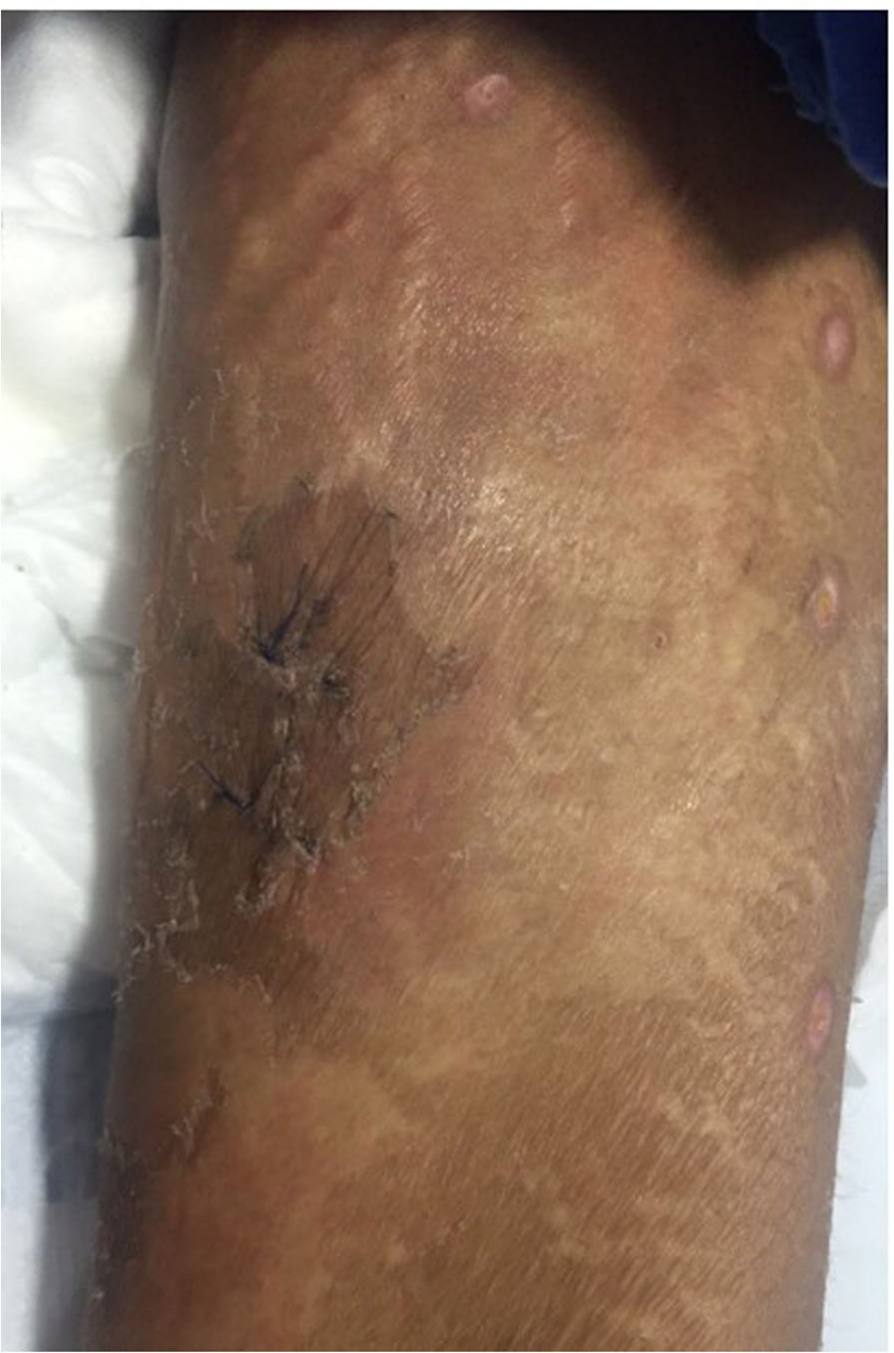
Proximal right thigh showed resolution of indurated plaque after 8 days of treatment for *Legionella*, biopsy stiches, healed scar of varicella lesions, and whitish striae

We describe a case of disseminated extrapulmonary legionellosis in an immunocompromised 38-year-old Thai woman. The patient was diagnosed in 2002 with systemic lupus erythematosus (SLE) with fever, polyarthritis, oral ulcer, alopecia, and proteinuria. Since then, she has been treated with prednisolone with azathioprine. She achieved clinical remission but remained on prednisolone (5 mg daily) and azathioprine (50 mg daily) for 13 years. In August 2015, 3 months prior to admission, she suffered from cramping abdominal pain, watery diarrhea two or three times daily, and low-grade fever. She was diagnosed with enteritis and treated with ceftriaxone without clinical improvement. The dose of immunosuppressive medication was increased to prednisolone 45 mg daily and hydroxychloroquine 400 mg daily. In September 2015, 2 months prior to admission, she developed proximal muscle weakness with low-grade fever. She was diagnosed with myasthenia gravis and received treatment with pyridostigmine (Mestinon™) 240 mg daily. She remained weak and lost significant weight because of poor appetite and diarrhea. She was admitted to her local hospital in October 2015 for intravenous fluid hydration and pyridostigmine was discontinued because of diarrhea. As her condition was becoming increasingly compromised with high-grade fever, generalized vesicular rash, and proximal muscle weakness, she was referred to our hospital in November, 2015. She did not recall any exposure to potentially contaminated water or animals. She worked as a school teacher. Upon admission, her temperature was 39 °C, heart rate 100 beats/min, and respiratory rate 20 breaths/min. Blood pressure was 90/60 mmHg. Physical examination revealed a cachectic woman with mild pale conjunctivae and anicteric sclerae. Skin examination showed generalized discrete erythematous papules and macules with dry necrotic crust on the scalp, facial area, trunk and extremities. She also had plaques measuring 15 × 15 cm on both inner thighs (Fig. 1). Abdominal examination showed mild tenderness and distension. The examination did not reveal any cardiac or pulmonary findings. Neurological examination revealed ptosis in both eyes, proximal muscle weakness (grade IV) of all extremities, but normal sensation and tendon reflexes. Laboratory data shown in Table 1. Skin biopsy of the crusted lesion revealed varicella zoster virus from polymerase chain reaction (PCR). She was diagnosed with varicella zoster virus infection. At admission, plasma cytomegalovirus (CMV) viral load (Cobas® Taqman amplicon) was 363,000 copies/mm^3^. She received intravenous ganciclovir injection with adjuvant granulocyte colony-stimulating factor for leukopenia. The timeline of the patient’s illness is illustrated in Additional file 1. She was also treated empirically for skin and soft tissue infection with piperacillin/tazobactam (12 days), and then meropenem (5 days) and then cefepime (5 days), without any clinical response. Further investigations, computed tomography of the abdomen showed a long segment of jejunal wall thickening and mild rectal wall thickening. Colonoscopy revealed generalized edematous mucosa of the colon without ulceration, and random biopsy was negative. She was diagnosed with CMV syndrome with suspected CMV jejunitis, which later improved with ganciclovir therapy. She was also diagnosed with myasthenia gravis by electromyography, nerve conduction velocity, and presence of acetylcholine receptor antibody. Later on, she developed chest pain and shortness of breath. Computed tomography of the chest revealed bilateral pleural effusion and small pericardial effusion. Echocardiography revealed impaired left ventricular systolic function with 40% ejection fraction along with global hypokinesia. She was diagnosed with lupus myocarditis, and treated with a 5-day course of intravenous immunoglobulin (0.4 g/kg/day) and 5 mg/day intravenous dexamethasone. During her hospitalization for 21 days, she remained febrile with a maximum temperature of 38.5–39 °C, despite the previously mentioned therapy. At that time, she had worsening pain in both thighs at the site of the plaques. Magnetic resonance imaging of both lower extremities revealed diffuse enhancing, hyperintense T2 signals in the muscles at the pelvis at both thighs and legs, with diffuse muscle atrophy and swelling of the skin and subcutaneous tissue (Fig. 2a, b). Multiple subcutaneous biopsy specimens were taken from both thighs (site of skin lesions) showed suppurative panniculitis (Fig. 3) and presence of Gram-negative bacilli. Acid-fast and Gomori methenamine stains were negative. Tissue biopsies for aerobic microorganisms showed no growth. Bacterial broad-range 16S ribosomal RNA sequencings revealed *L. pneumophila* (99% similarity to *L. pneumophila* consensus sequence). Culture for fungi and mycobacteria was negative. Her antimicrobial regimen was changed to intravenous azithromycin, and fever subsided within 5 days. Her thigh lesions gradually improved over the first week of therapy (Fig. 4). She was diagnosed with disseminated *L. pneumophila* infection resulting in panniculitis, myositis and myocarditis. She received intravenous azithromycin for 21 days. Oral azithromycin and ciprofloxacin were continued for 3 months to ensure eradication of the organism from our immunosuppressed patient. She received intravenous ganciclovir until the clearance of CMV viremia (total of 48 days), and then switched to oral valganciclovir maintenance therapy. She underwent physical rehabilitation and was discharged after 64 days hospitalization. Clinically, she is doing well at 1-year follow-up. She did not have any further tests done as follow-up proved successful clinical resolution and eradication of *Legionella* infection.

**Table 1:** Laboratory data on admission

Parameter	Recorded value	Standard value
White blood cell count	1,860 cells/mm^3^	4,500–7,500 cells/mm^3^
Neutrophils	83 %	
Lymphocytes	14 %	
Hemoglobin	10.8 g/dL	11.3–15.2 g/dL
Hematocrit	32.4 %	36–45 %
Platelet count	179,000 cells/mm^3^	130,000–350,000 cells/mm^3^
Total protein	33 g/L	69-84 g/L
Albumin	12.3 g/L	39–51 g/L
Total bilirubin	0.6 mg/dL	0.2–1.2 mg/dL
Direct billirubin	0.3 mg/dL	0.1–0.3 mg/dL
Aspartate aminotransferase	23 U/L	11–30 U/L
Alanine aminotransferase	34 U/L	4–30 U/L
Alkaline phosphatase	57 U/L	44–147 U/L
Blood urea nitrogen	9.0 mg/dL	8–20 mg/dL
Creatinine	0.32 mg/dL	0.63–1.03 mg/dL

## Discussion

Here, we describe a patient with SLE and myasthenia gravis who suffered multiple infectious complications including varicella, CMV syndrome/jejunitis and *Legionella* panniculitis with possible dissemination (myositis and myocarditis). Panniculitis cause by *Legionella* has not been reported in the literature. Our initial presumptive diagnosis of panniculitis was lupus panniculitis or CMV related panniculitis. However, our patient did not improve during the course of treatment for both diseases. The diagnosis of lupus panniculitis should not be assumed in SLE patients, given that immunosuppressive drugs are a predisposing factor for infective panniculitis, and both conditions require different treatment strategies [10]. Infective panniculitis has been described in association with many infectious agents including bacteria (*Nocardia* spp. and *Actinomyces* spp.), mycobacteria, fungi and parasites [11]. Anatomical pathology is the key to distinguishing the etiology of panniculitis by the type of white blood cell infiltrates. Lupus panniculitis presents with lymphocytic infiltrates with or without vasculitis. CMV panniculitis must have evidence of viral cytopathic changes with the characteristic owl’s eye inclusion bodies. Our patient had neutrophilic panniculitis that suggested infective panniculitis. Gram staining showed intracellular Gram-negative bacilli that failed to grow on routine culture media, and they were identified as *L. pneumophila* by molecular methods. It was difficult to distinguish whether the panniculitis resulted from primary inoculation or secondary hematogenous spread of *Legionella*. Several features, such as prolonged fever despite broad-spectrum beta-lactam antibiotics, myositis and myocarditis, suggested disseminated *Legionella* infection. Myositis has been reported in one patient with pneumonia [12]. The etiology of myocarditis in our patient remains debatable, and whether it resulted from lupus or *Legionella* infection. Myocardial biopsy was not performed because of the excessive risk in our case, and therapy for both lupus and *Legionella* was initiated at the same time. Follow-up echocardiography showed normal cardiac function. *Legionella* myocarditis has been reported as a complication of Legionnaires’ disease [13, 14]. Only one adult patient developed perimyocarditis due to *L. pneumophila* in the absence of respiratory involvement, which resulted in multiorgan failure [1]. The atypical multiorgan involvement of *Legionella* infection in our patient could have resulted from immunosuppression that led to lymphopenia and cell-mediated immunodeficiency. Our patient did not have evidence of pneumonia at any time point before or during hospitalization.

Cutaneous and subcutaneous *Legionella* infection is rare and mostly occurs in immunosuppressed patients, as described previously [5]. Primary extrapulmonary infection of skin/subcutaneous tissue (cellulitis, multiple subcutaneous abscesses, and tenosynovitis) by *L. pneumophila* led to disseminated infection (pneumonia and respiratory failure) in an immunocompromised liver transplant recipient [15]. The diagnosis was made from culture of bronchoalveolar lavage fluid and later, *Legionella* was isolated from the abscesses using special culture media and was confirmed by molecular methods [15]. This highlights the challenging aspect in the diagnosis of *Legionella* infection, as it is a facultative Gram-negative aerobic bacillus that resides within tissue and alveolar macrophages, and it requires specialized media [16]. Non-culture-based diagnostic methods include urinary antigen tests for *L. pneumophila*, which is a rapid diagnostic tool; however, these tests are limited to the detection of *L. pneumophila* serogroup 1, *L. micdadei* and *Legionella longbeachae* [17].

The diagnosis of extrapulmonary *Legionella* infection relies on clinician alertness and a good level of cooperation with the microbiology laboratory, as *Legionella* species must be grown on special media. In our setting we did not have the selective media, *Legionella* urinary antigen and antibody to detect *Legionella*, or direct fluorescent antibody against *Legionella*. The diagnosis in our patient was made by 16S rRNA gene sequencing method. Initiation of appropriate treatment is critical because delay is associated with increased mortality in *Legionella* pneumonia [18]. Effective antimicrobial treatment of legionellosis includes antibiotics that achieve therapeutic intracellular concentrations within macrophages, such as the macrolides, fluoroquinolones, and cyclin families [16]. Azithromycin or levofloxacin are commonly used to treat *Legionella* infection [16, 19]. Optimal treatment duration for cutaneous legionellosis has not been established. Most patients with *Legionella* pneumonia are successfully treated with a 7-to 14-day course of antibiotics. Disseminated legionellosis requires longer duration of therapy, although the duration is not well defined. Immunocompromised patients with cutaneous legionellosis may require 3 weeks of treatment [5, 16].

## Conclusion

*Legionella* infection may cause extrapulmonary manifestations involving skin/subcutaneous and muscle that leads to dissemination, especially in immunocompromised patients. Infective panniculitis caused by *Legionella* should be considered in the differential diagnosis of skin/subcutaneous infection that fails to respond to beta-lactam antibiotics. The diagnosis of extrapulmonary *Legionella* infection is challenging because special culture media are required. In our case, the diagnosis was based on 16S rRNA gene sequencing method. Clinicians should be aware of extrapulmonary manifestation of legionellosis in the absence of pneumonia.

## Additional file

Additional file 1: The timeline of the patient’s illness. The patient visited the local hospital with low-grade fever and diarrhea for 3 months. She had varicella and cytomegalovirus syndrome with jejunitis. She had persistence high-grade fever and panniculitis despite broad-spectrum antibiotics. She developed myocarditis. She recovered after receiving specific therapy. (DOCX 70 kb)

## Abbreviations

PCR: Polymerase chain reaction
rRNA: ribosomal ribonucleic acid
