# Supplementary material for: Disseminated extrapulmonary Legionella pneumophila infection presenting with panniculitis: case report and literature review
Source: BMC Infect Dis. 2018 Sep 17;18:467. doi: 10.1186/s12879-018-3378-0 (PMC6142325; doi:10.1186/s12879-018-3378-0)
Supplement: Supplementary file 1 — The timeline of the patient’s illness. The patient visited the local hospital with low-grade fever and diarrhea for 3 months. She had varicella and cytomegalovirus syndrome with jejunitis. She had persistence high-grade fever and panniculitis despite broad-spectrum antibiotics. She developed myocarditis. She recovered after receiving specific therapy. (DOCX 70 kb) [file 12879_2018_3378_MOESM1_ESM.docx]

Proximal muscle weakness with a low-grade fever for one month

CT abdomen: long segment of jejunal wall thickening and mild rectal wall thickening.

October 22, 2015

Admitted to Hospital

Empirical Antibiotics Therapy

Meropenem for 3 days then

Piperacillin/tazobactam for 13 days then Cefepime for 6 days.

Prednisolone 15 mg/day

Discharged from hospital with oral ciprofloxacin and azithromycin for three months.

Complete resolution of symptom.

Treatment: intravenous ganciclovir for 48 days

October 29, 2015

Diagnosis: Legionella panniculitis, suspect dissemination (myositis, myocarditis)

Treatment : intravenous azithromycin for 21 days

Treatment : IVIG for 5 days IV. Dexamethasone 5 mg daily

November 11, 2015

Subcutaneous biopsy report of 16S rRNA *L. pneumophila*

Diagnosis : Infective panniculitis, myositis

November 6, 2015

MRI of thigh: diffuse enhancing, hyperintense T2 signal of the muscles of both thighs and legs with diffuse muscles atrophy and swelling of the skin and subcutaneous tissue

October 23, 2015

October 25, 2015

October 26, 2015

Diagnosis: Lupus myocarditis

Echocardiogram revealed impair LVEF of 40%, global hypokinesia.

Subcutaneous biopsy of the both thigh at the site of skin lesion showed suppurative panniculitis and Gram-negative bacilli

Diagnosis: CMV syndrome with suspected CMV jejunitis

October 24, 2015

38-year-old Thai woman, SLE and Myasthenia Gravis

Diagnosis: Enteritis

Cramping abdominal pain, watery diarrhea 2-3 times a day and a low-grade fever for 3 months

Plasma CMV viral load 363,000 copies/mm^3^

Diagnosis: Varicella, Panniculitis, SLE, Myasthenia gravis

High-grade fever with chills, and generalized vesicular rash for one week

PE : generalized discrete erythematous papule and macule with dry necrotic crust

Multiple indurated erythematous plaque at both inner thigh size 15 x 15 cm.

Diagnosis: Myasthenia gravis

Treatment: pyridostigmine (mestinon™) 240 mg daily

Treatment: intravenous hydration

Treatment: prednisolone 45 mg daily and hydroxychloroquine 400 mg daily
